# Cyclo­linopeptide A methanol solvate

**DOI:** 10.1107/S1600536809026841

**Published:** 2009-07-18

**Authors:** J. Wilson Quail, J. Shen, M. J. T. Reaney, R. Sammynaiken

**Affiliations:** a Saskatchewan Structural Sciences Centre, University of Saskatchewan, 110 Science Place, Saskatoon, Saskatchewan S7N 5C9, Canada; b Department of Food and Bioproduct Sources, College of Agriculture and Bioresources, University of Saskatchewan, Saskatoon, Saskatchewan S7N 5A8, Canada

## Abstract

Crystals of the title compound, C_57_H_85_N_9_O_9_·CH_4_O, the methanol solvate of a nine peptide polypeptide, *cyclo*-(Pro-Pro-Phe-Phe-Leu-Ile-Ile-Leu-Val), were obtained after separation of the cyclic peptide from flax oil. The cyclo­linopeptide A (CLP-A) mol­ecules are linked in chains along the *a* axis by N—H⋯O hydrogen bonds. Each methanol O atom is hydrogen bonded to one O atom and two N—H groups in the same CLP-A mol­ecule. There are a total of eight hydrogen bonds in each CLP-A–MeOH unit.

## Related literature

For the isolation of CLP-A, see: Kaufmann & Tobschirbel (1959 ▶). For its synthesis and absolute configuration, see: Prox & Weygand (1966 ▶). For the crystal structure of the 2-propanol solvate, see: Di Blasio *et al.* (1989 ▶). For NMR studies and the crystal structure of a solvate of unstated composition of CLP-A, see: Matsumoto *et al.* (2002 ▶). For the cytoprotective ability of CLP-A, see: Kessler *et al.* (1986 ▶).
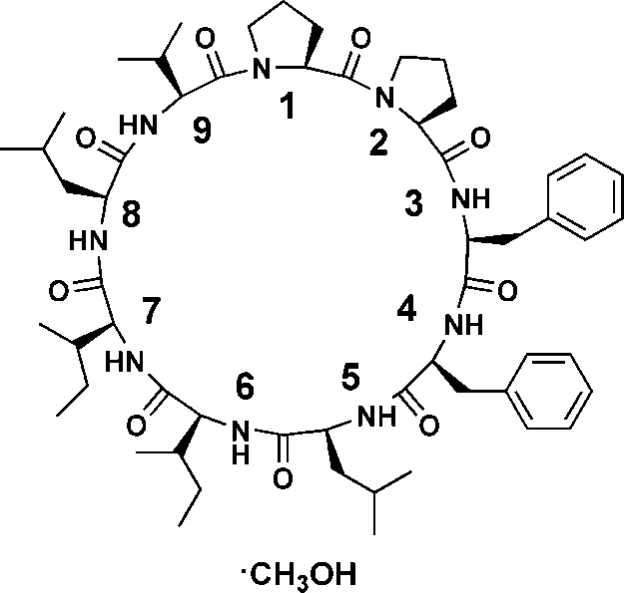


## Experimental

### 

#### Crystal data

C_57_H_85_N_9_O_9_·CH_4_O
*M* *_r_* = 1072.38
Orthorhombic, 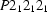

*a* = 9.8650 (4) Å
*b* = 22.6135 (5) Å
*c* = 26.5723 (10) Å
*V* = 5927.8 (4) Å^3^
*Z* = 4
Mo *K*α radiation
μ = 0.08 mm^−1^
*T* = 173 K
0.20 × 0.05 × 0.05 mm

#### Data collection

Nonius KappaCCD diffractometer
Absorption correction: none
10352 measured reflections
5817 independent reflections
3987 reflections with *I* > 2σ(*I*)
*R*_int_ = 0.070

#### Refinement

*R*[*F* ^2^ > 2σ(*F* ^2^)] = 0.055
*wR*(*F* ^2^) = 0.110
*S* = 1.04
5817 reflections
707 parameters
H-atom parameters constrained
Δρ_max_ = 0.18 e Å^−3^
Δρ_min_ = −0.18 e Å^−3^

### 

Data collection: *COLLECT* (Nonius, 1998 ▶); cell refinement: *SCALEPACK* (Otwinowski & Minor, 1997 ▶); data reduction: *DENZO* (Otwinowski & Minor, 1997 ▶) and *SCALEPACK*; program(s) used to solve structure: *SIR97* (Altomare *et al.*, 1999 ▶); program(s) used to refine structure: *SHELXTL* (Sheldrick, 2008 ▶); molecular graphics: *ORTEP-3 for Windows* (Farrugia, 1997 ▶); software used to prepare material for publication: *SHELXTL*.

## Supplementary Material

Crystal structure: contains datablocks I, global. DOI: 10.1107/S1600536809026841/pv2179sup1.cif

Structure factors: contains datablocks I. DOI: 10.1107/S1600536809026841/pv2179Isup2.hkl

Additional supplementary materials:  crystallographic information; 3D view; checkCIF report

## Figures and Tables

**Table 1:** Hydrogen-bond geometry (Å, °)

*D*—H⋯*A*	*D*—H	H⋯*A*	*D*⋯*A*	*D*—H⋯*A*
N3—H3⋯O10	0.88	2.09	2.912 (5)	154
N4—H4⋯O10	0.88	2.28	3.139 (5)	166
N4—H4⋯O9	0.88	2.51	3.103 (5)	125
N5—H5⋯O3	0.88	2.18	2.935 (5)	143
N6—H6⋯O8^i^	0.88	2.49	3.208 (5)	139
N7—H7⋯O4	0.88	2.50	3.266 (5)	145
N8—H8⋯O5	0.88	2.11	2.971 (5)	165
N9—H9⋯O4	0.88	2.13	2.975 (5)	161
O10—H10⋯O9	0.84	1.91	2.698 (4)	156

Symmetry code: (i) 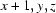.

**Fig. 1.**
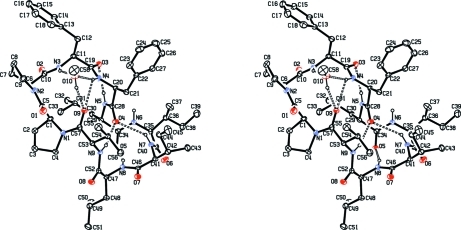
The stereo molecular structure of the title compound, with atom labels and 20% probability displacement ellipsoids for non-H atoms. Only H atoms on N or O atoms are included. The methanol molecule (C58—O10) has the C atom pointing toward the viewer.

## supplementary crystallographic information

### Experimental

The title compound was extracted from flax oil by successive elution of a mixture of 700 ml of flax oil and 700 ml of 5% ethyl acetate in hexane. Fractions containing several cyclicpeptides were collected and dried by rotary evaporation. Further separation was achieved by using HPLC and acetonitrile/water solvent gradient. Each cyclicpeptide fraction was collectedand identified by NMR and MS. CLP-A was crystallized by dissolving it in minimum amount of methanol and then adding a few drops of water to the solution. After two days of slow evaporation of the methanol at room temperature, suitable crystals were harvested for crystallographic studies.

### Refinement

H atoms bonded to N atoms were located in a difference map and then positioned geometrically with *U*_iso_ constrained to be 1.2 times *U*_eq_(N).and the bond length constrained to 0.88 Å. Other H atoms were positioned geometrically and refined using a riding model (including free rotation for methyl groups with C—H = 0.95–0.99 Å and with *U*_iso_(H) = 1.2(1.5 for methyl groups) times *U*_eq_(C).

The chirality of the amino acids can not be determined from the anomalous dispersion, but the relative chirality is clearly all the same. The structure has no atom with *Z*>8. With Mo radiation and low resolution data, it is impossible to determine the absolute chirality from these data. Based on the synthesis of the molecule by Prox & Weygand (1966), we have assumed that all amino acids are S. All Friedel mates (4536) were merged in the refinement.

### Crystal data

**Table:**

C_57_H_85_N_9_O_9_·CH_4_O	*F*(000) = 2320
*M**_r_* = 1072.38	*D*_x_ = 1.202 Mg m^−^^3^
Orthorhombic, *P*2_1_2_1_2_1_	Mo *K*α radiation, λ = 0.71073 Å
Hall symbol: P 2ac 2ab	Cell parameters from 5719 reflections
*a* = 9.8650 (4) Å	θ = 1.0–25.0°
*b* = 22.6135 (5) Å	µ = 0.08 mm^−^^1^
*c* = 26.5723 (10) Å	*T* = 173 K
*V* = 5927.8 (4) Å^3^	Rod, colourless
*Z* = 4	0.20 × 0.05 × 0.05 mm

### Data collection

**Table:**

Nonius KappaCCD diffractometer	3987 reflections with *I* > 2σ(*I*)
Radiation source: fine-focus sealed tube	*R*_int_ = 0.070
horizonally mounted graphite crystal	θ_max_ = 25.0°, θ_min_ = 2.5°
Detector resolution: 9 pixels mm^-1^	*h* = −11→11
φ scans and ω scans with κ offsets	*k* = −26→26
10352 measured reflections	*l* = −31→31
5817 independent reflections	

### Refinement

**Table:**

Refinement on *F*^2^	Hydrogen site location: inferred from neighbouring sites
Least-squares matrix: full	H-atom parameters constrained
*R*[*F*^2^ > 2σ(*F*^2^)] = 0.055	*w* = 1/[σ^2^(*F*_o_^2^) + (0.0289*P*)^2^ + 2.503*P*] where *P* = (*F*_o_^2^ + 2*F*_c_^2^)/3
*wR*(*F*^2^) = 0.110	(Δ/σ)_max_ < 0.001
*S* = 1.04	Δρ_max_ = 0.18 e Å^−^^3^
5817 reflections	Δρ_min_ = −0.18 e Å^−^^3^
707 parameters	Extinction correction: *SHELXTL* (Sheldrick, 2008), Fc^*^=kFc[1+0.001xFc^2^λ^3^/sin(2θ)]^-1/4^
0 restraints	Extinction coefficient: 0.0040 (3)
Primary atom site location: structure-invariant direct methods	Absolute structure: syn
Secondary atom site location: difference Fourier map	

### Special details

**Table:**

Geometry. All e.s.d.'s (except the e.s.d. in the dihedral angle between two l.s. planes) are estimated using the full covariance matrix. The cell e.s.d.'s are taken into account individually in the estimation of e.s.d.'s in distances, angles and torsion angles; correlations between e.s.d.'s in cell parameters are only used when they are defined by crystal symmetry. An approximate (isotropic) treatment of cell e.s.d.'s is used for estimating e.s.d.'s involving l.s. planes.
Refinement. Refinement of *F*^2^ against ALL reflections. The weighted *R*-factor *wR* and goodness of fit *S* are based on *F*^2^, conventional *R*-factors *R* are based on *F*, with *F* set to zero for negative *F*^2^. The threshold expression of *F*^2^ > σ(*F*^2^) is used only for calculating *R*-factors(gt) *etc*. and is not relevant to the choice of reflections for refinement. *R*-factors based on *F*^2^ are statistically about twice as large as those based on *F*, and *R*- factors based on ALL data will be even larger.

### Fractional atomic coordinates and isotropic or equivalent isotropic displacement parameters (Å^2^)

**Table:**

	*x*	*y*	*z*	*U*_iso_*/*U*_eq_	
O1	0.6490 (4)	0.14168 (15)	0.41141 (12)	0.0430 (9)	
O2	1.1761 (4)	0.24064 (13)	0.42479 (13)	0.0478 (9)	
O3	1.3224 (3)	0.14204 (15)	0.30740 (11)	0.0416 (8)	
O4	0.9549 (3)	0.15024 (13)	0.20281 (11)	0.0347 (8)	
O5	1.0026 (3)	0.27819 (12)	0.13135 (10)	0.0339 (8)	
O6	1.0140 (4)	0.20528 (15)	0.00902 (12)	0.0473 (9)	
O7	0.6008 (3)	0.14060 (13)	0.09504 (11)	0.0378 (8)	
O8	0.4485 (3)	0.20492 (14)	0.19539 (12)	0.0396 (8)	
O9	0.8048 (3)	0.11313 (13)	0.31070 (11)	0.0366 (8)	
N1	0.6840 (4)	0.19658 (15)	0.32071 (13)	0.0315 (9)	
N2	0.8407 (4)	0.17833 (15)	0.44576 (13)	0.0326 (9)	
N3	1.0975 (4)	0.14799 (15)	0.40928 (13)	0.0301 (9)	
H3	1.0270	0.1242	0.4105	0.036*	
N4	1.1175 (4)	0.09880 (15)	0.30765 (13)	0.0311 (9)	
H4	1.0487	0.0865	0.3260	0.037*	
N5	1.1410 (4)	0.19582 (14)	0.23273 (12)	0.0293 (9)	
H5	1.2181	0.1928	0.2492	0.035*	
N6	1.1765 (4)	0.21291 (14)	0.12955 (12)	0.0306 (9)	
H6	1.2336	0.1916	0.1474	0.037*	
N7	0.9621 (4)	0.15497 (15)	0.08001 (13)	0.0297 (9)	
H7	0.9907	0.1431	0.1097	0.036*	
N8	0.7432 (4)	0.21286 (14)	0.11963 (13)	0.0300 (9)	
H8	0.8267	0.2266	0.1198	0.036*	
N9	0.6568 (4)	0.16721 (15)	0.21162 (13)	0.0303 (9)	
H9	0.7424	0.1668	0.2022	0.036*	
C1	0.7682 (5)	0.21527 (17)	0.36348 (15)	0.0317 (11)	
H1	0.8661	0.2166	0.3538	0.038*	
C2	0.7145 (5)	0.27780 (19)	0.37430 (18)	0.0396 (12)	
H2A	0.7217	0.2873	0.4106	0.047*	
H2B	0.7653	0.3078	0.3548	0.047*	
C3	0.5667 (5)	0.2754 (2)	0.35773 (18)	0.0413 (13)	
H3A	0.5092	0.2566	0.3838	0.050*	
H3B	0.5313	0.3154	0.3504	0.050*	
C4	0.5729 (5)	0.23786 (19)	0.31047 (17)	0.0349 (12)	
H4A	0.4865	0.2165	0.3050	0.042*	
H4B	0.5926	0.2624	0.2805	0.042*	
C5	0.7468 (5)	0.17461 (19)	0.40867 (17)	0.0329 (11)	
C6	0.9504 (5)	0.22168 (18)	0.45025 (16)	0.0318 (11)	
H6A	0.9202	0.2601	0.4354	0.038*	
C7	0.9657 (6)	0.2288 (2)	0.50758 (17)	0.0472 (14)	
H7A	1.0596	0.2399	0.5167	0.057*	
H7B	0.9025	0.2591	0.5208	0.057*	
C8	0.9302 (6)	0.1678 (2)	0.52744 (18)	0.0493 (15)	
H8A	1.0090	0.1408	0.5250	0.059*	
H8B	0.9005	0.1699	0.5630	0.059*	
C9	0.8159 (6)	0.1470 (2)	0.49388 (16)	0.0444 (13)	
H9A	0.8190	0.1036	0.4892	0.053*	
H9B	0.7267	0.1580	0.5082	0.053*	
C10	1.0851 (5)	0.20366 (19)	0.42662 (16)	0.0322 (11)	
C11	1.2247 (5)	0.12615 (18)	0.38849 (15)	0.0297 (11)	
H11	1.2966	0.1552	0.3982	0.036*	
C12	1.2646 (5)	0.06659 (18)	0.41182 (16)	0.0349 (12)	
H12A	1.1928	0.0372	0.4047	0.042*	
H12B	1.3496	0.0525	0.3960	0.042*	
C13	1.2848 (5)	0.07110 (18)	0.46802 (16)	0.0333 (11)	
C14	1.3965 (6)	0.1017 (2)	0.48615 (18)	0.0409 (13)	
H14	1.4596	0.1186	0.4633	0.049*	
C15	1.4166 (6)	0.1079 (2)	0.53792 (19)	0.0485 (14)	
H15	1.4920	0.1298	0.5500	0.058*	
C16	1.3284 (7)	0.0826 (2)	0.5714 (2)	0.0557 (17)	
H16	1.3433	0.0862	0.6065	0.067*	
C17	1.2180 (7)	0.0521 (2)	0.5536 (2)	0.0598 (17)	
H17	1.1556	0.0349	0.5765	0.072*	
C18	1.1972 (6)	0.0460 (2)	0.5022 (2)	0.0485 (14)	
H18	1.1212	0.0243	0.4904	0.058*	
C19	1.2238 (5)	0.12317 (19)	0.33097 (16)	0.0314 (11)	
C20	1.1107 (5)	0.09171 (17)	0.25296 (15)	0.0269 (10)	
H20	1.2049	0.0842	0.2406	0.032*	
C21	1.0242 (5)	0.03878 (16)	0.23827 (17)	0.0329 (11)	
H21A	0.9398	0.0392	0.2584	0.039*	
H21B	0.9986	0.0424	0.2024	0.039*	
C22	1.0956 (5)	−0.01974 (17)	0.24623 (16)	0.0298 (11)	
C40	1.0429 (5)	0.18986 (18)	0.05201 (17)	0.0326 (11)	
C23	1.0602 (6)	−0.0571 (2)	0.28522 (18)	0.0439 (13)	
H23	0.9903	−0.0462	0.3080	0.053*	
C24	1.1268 (6)	−0.1108 (2)	0.2911 (2)	0.0524 (15)	
H24	1.1013	−0.1366	0.3177	0.063*	
C25	1.2290 (6)	−0.1269 (2)	0.2589 (2)	0.0529 (15)	
H25	1.2744	−0.1636	0.2633	0.064*	
C26	1.2657 (6)	−0.0897 (2)	0.2200 (2)	0.0483 (14)	
H26	1.3366	−0.1006	0.1976	0.058*	
C27	1.1989 (5)	−0.03658 (19)	0.21373 (17)	0.0380 (12)	
H27	1.2239	−0.0112	0.1868	0.046*	
C28	1.0596 (5)	0.14826 (19)	0.22772 (15)	0.0284 (11)	
C29	1.1011 (5)	0.25230 (17)	0.21081 (15)	0.0279 (11)	
H29	1.0096	0.2627	0.2244	0.033*	
C30	1.1993 (5)	0.30132 (18)	0.22619 (15)	0.0323 (11)	
H30A	1.1669	0.3389	0.2113	0.039*	
H30B	1.2888	0.2925	0.2111	0.039*	
C31	1.2193 (5)	0.31099 (18)	0.28272 (16)	0.0348 (12)	
H31	1.2666	0.2755	0.2968	0.042*	
C32	1.3105 (6)	0.3640 (2)	0.28986 (19)	0.0497 (14)	
H32A	1.3969	0.3572	0.2726	0.075*	
H32B	1.3271	0.3701	0.3259	0.075*	
H32C	1.2667	0.3992	0.2757	0.075*	
C33	1.0869 (6)	0.3191 (2)	0.31117 (18)	0.0483 (14)	
H33A	1.1061	0.3247	0.3470	0.072*	
H33B	1.0302	0.2838	0.3068	0.072*	
H33C	1.0391	0.3538	0.2981	0.072*	
C34	1.0895 (5)	0.24901 (18)	0.15363 (16)	0.0279 (11)	
C35	1.1790 (5)	0.20796 (19)	0.07441 (15)	0.0327 (11)	
H35	1.2018	0.2479	0.0607	0.039*	
C36	1.2942 (5)	0.16549 (19)	0.05862 (17)	0.0380 (12)	
H36	1.3796	0.1816	0.0736	0.046*	
C37	1.2770 (6)	0.1035 (2)	0.0797 (2)	0.0570 (16)	
H37A	1.2546	0.1058	0.1155	0.085*	
H37B	1.3617	0.0813	0.0754	0.085*	
H37C	1.2038	0.0832	0.0617	0.085*	
C38	1.3130 (6)	0.1670 (2)	0.00099 (18)	0.0566 (16)	
H38A	1.2324	0.1490	−0.0150	0.068*	
H38B	1.3176	0.2088	−0.0101	0.068*	
C39	1.4375 (6)	0.1354 (3)	−0.0172 (2)	0.0685 (18)	
H39A	1.5183	0.1544	−0.0031	0.103*	
H39B	1.4413	0.1372	−0.0540	0.103*	
H39C	1.4341	0.0940	−0.0064	0.103*	
C41	0.8284 (5)	0.13627 (18)	0.06258 (16)	0.0302 (11)	
H41	0.8180	0.1522	0.0277	0.036*	
C42	0.8155 (5)	0.06915 (17)	0.05847 (16)	0.0334 (11)	
H42	0.7187	0.0600	0.0504	0.040*	
C43	0.9024 (6)	0.04506 (19)	0.01514 (17)	0.0432 (13)	
H43A	0.8737	0.0634	−0.0165	0.065*	
H43B	0.8909	0.0021	0.0129	0.065*	
H43C	0.9979	0.0543	0.0214	0.065*	
C44	0.8494 (6)	0.03857 (19)	0.10812 (17)	0.0430 (13)	
H44A	0.8101	0.0619	0.1361	0.052*	
H44B	0.9490	0.0383	0.1125	0.052*	
C45	0.7974 (7)	−0.0249 (2)	0.1119 (2)	0.0662 (19)	
H45A	0.6985	−0.0251	0.1083	0.099*	
H45B	0.8222	−0.0414	0.1447	0.099*	
H45C	0.8381	−0.0488	0.0851	0.099*	
C46	0.7138 (5)	0.16320 (19)	0.09380 (16)	0.0293 (11)	
C47	0.6373 (5)	0.24396 (18)	0.14739 (16)	0.0296 (11)	
H47	0.6852	0.2751	0.1674	0.036*	
C48	0.5339 (5)	0.27708 (18)	0.11482 (17)	0.0349 (12)	
H48A	0.5687	0.2789	0.0799	0.042*	
H48B	0.4485	0.2541	0.1142	0.042*	
C49	0.5025 (5)	0.33970 (18)	0.13241 (17)	0.0381 (12)	
H49	0.4957	0.3395	0.1700	0.046*	
C50	0.6146 (6)	0.3831 (2)	0.1172 (2)	0.0574 (16)	
H50A	0.6124	0.3891	0.0807	0.086*	
H50B	0.7030	0.3670	0.1270	0.086*	
H50C	0.6001	0.4210	0.1342	0.086*	
C51	0.3655 (6)	0.3595 (2)	0.1108 (2)	0.0532 (15)	
H51A	0.3689	0.3581	0.0740	0.080*	
H51B	0.3462	0.4001	0.1217	0.080*	
H51C	0.2939	0.3331	0.1230	0.080*	
C52	0.5702 (5)	0.20273 (19)	0.18643 (16)	0.0318 (11)	
C53	0.6163 (5)	0.12948 (18)	0.25350 (15)	0.0299 (11)	
H53	0.5214	0.1403	0.2630	0.036*	
C54	0.6182 (5)	0.06326 (19)	0.24054 (16)	0.0352 (12)	
H54	0.7131	0.0513	0.2321	0.042*	
C55	0.5690 (6)	0.0265 (2)	0.28561 (19)	0.0494 (14)	
H55A	0.4757	0.0377	0.2940	0.074*	
H55B	0.6278	0.0339	0.3146	0.074*	
H55C	0.5720	−0.0156	0.2770	0.074*	
C56	0.5271 (5)	0.05127 (19)	0.19503 (17)	0.0418 (13)	
H56A	0.5341	0.0095	0.1855	0.063*	
H56B	0.5560	0.0761	0.1668	0.063*	
H56C	0.4329	0.0605	0.2037	0.063*	
C57	0.7083 (5)	0.14515 (19)	0.29755 (15)	0.0316 (11)	
O10	0.9112 (4)	0.05125 (13)	0.38798 (13)	0.0459 (9)	
H10	0.8568	0.0675	0.3680	0.069*	
C58	0.8484 (7)	0.0016 (2)	0.4104 (2)	0.077 (2)	
H58A	0.9140	−0.0190	0.4318	0.115*	
H58B	0.8163	−0.0253	0.3840	0.115*	
H58C	0.7714	0.0147	0.4309	0.115*	

### Atomic displacement parameters (Å^2^)

**Table:**

	*U*^11^	*U*^22^	*U*^33^	*U*^12^	*U*^13^	*U*^23^
O1	0.036 (2)	0.056 (2)	0.0375 (19)	−0.0097 (19)	0.0011 (17)	0.0004 (17)
O2	0.039 (2)	0.0397 (18)	0.065 (2)	−0.0085 (18)	0.000 (2)	−0.0096 (17)
O3	0.028 (2)	0.065 (2)	0.0321 (18)	−0.0092 (18)	0.0015 (17)	−0.0004 (16)
O4	0.025 (2)	0.0398 (17)	0.0397 (18)	−0.0002 (15)	−0.0053 (16)	0.0026 (15)
O5	0.035 (2)	0.0353 (16)	0.0312 (17)	0.0046 (16)	−0.0063 (16)	0.0005 (14)
O6	0.036 (2)	0.074 (2)	0.0314 (19)	−0.009 (2)	−0.0023 (17)	0.0084 (17)
O7	0.027 (2)	0.0432 (18)	0.043 (2)	0.0017 (17)	−0.0029 (17)	−0.0028 (16)
O8	0.024 (2)	0.0544 (19)	0.0400 (19)	0.0091 (17)	−0.0007 (16)	0.0018 (16)
O9	0.033 (2)	0.0461 (18)	0.0306 (18)	0.0125 (17)	−0.0018 (16)	0.0001 (15)
N1	0.027 (2)	0.040 (2)	0.027 (2)	0.0016 (19)	−0.0005 (18)	−0.0023 (17)
N2	0.027 (2)	0.042 (2)	0.029 (2)	−0.0045 (19)	−0.0007 (19)	0.0025 (17)
N3	0.027 (2)	0.0294 (19)	0.034 (2)	−0.0047 (17)	−0.0002 (18)	−0.0032 (17)
N4	0.028 (2)	0.039 (2)	0.027 (2)	−0.0071 (18)	−0.0003 (19)	−0.0032 (17)
N5	0.027 (2)	0.0325 (19)	0.028 (2)	0.0026 (18)	−0.0056 (18)	−0.0006 (16)
N6	0.031 (2)	0.032 (2)	0.029 (2)	0.0040 (18)	−0.0012 (18)	−0.0013 (16)
N7	0.028 (2)	0.034 (2)	0.027 (2)	0.0058 (18)	−0.0013 (18)	0.0046 (16)
N8	0.022 (2)	0.036 (2)	0.032 (2)	0.0002 (18)	−0.0001 (17)	−0.0021 (17)
N9	0.023 (2)	0.043 (2)	0.025 (2)	0.0054 (18)	0.0006 (17)	−0.0012 (17)
C1	0.024 (3)	0.040 (2)	0.031 (2)	0.000 (2)	−0.005 (2)	0.001 (2)
C2	0.042 (3)	0.043 (3)	0.035 (3)	0.004 (3)	−0.004 (2)	−0.002 (2)
C3	0.038 (3)	0.043 (3)	0.043 (3)	0.008 (2)	0.000 (3)	−0.004 (2)
C4	0.027 (3)	0.040 (2)	0.037 (3)	0.010 (2)	−0.001 (2)	−0.003 (2)
C5	0.028 (3)	0.038 (3)	0.032 (3)	0.001 (2)	−0.001 (2)	−0.004 (2)
C6	0.027 (3)	0.032 (2)	0.036 (3)	0.000 (2)	−0.004 (2)	−0.004 (2)
C7	0.049 (4)	0.059 (3)	0.033 (3)	0.001 (3)	−0.004 (3)	−0.011 (2)
C8	0.059 (4)	0.059 (3)	0.030 (3)	0.005 (3)	−0.007 (3)	−0.005 (2)
C9	0.054 (4)	0.049 (3)	0.029 (3)	0.000 (3)	0.001 (3)	0.007 (2)
C10	0.034 (3)	0.033 (2)	0.031 (2)	−0.006 (2)	−0.005 (2)	−0.005 (2)
C11	0.022 (3)	0.037 (2)	0.030 (2)	0.000 (2)	−0.002 (2)	0.003 (2)
C12	0.032 (3)	0.036 (2)	0.037 (3)	0.004 (2)	0.001 (2)	0.003 (2)
C13	0.031 (3)	0.037 (2)	0.032 (3)	0.006 (2)	−0.002 (2)	0.005 (2)
C14	0.035 (3)	0.051 (3)	0.037 (3)	0.007 (3)	0.001 (3)	0.007 (2)
C15	0.055 (4)	0.048 (3)	0.043 (3)	0.009 (3)	−0.008 (3)	−0.002 (2)
C16	0.088 (5)	0.042 (3)	0.037 (3)	0.017 (3)	0.001 (3)	0.001 (3)
C17	0.087 (5)	0.047 (3)	0.045 (3)	0.010 (3)	0.023 (4)	0.016 (3)
C18	0.046 (4)	0.042 (3)	0.057 (4)	−0.001 (3)	0.003 (3)	0.009 (3)
C19	0.028 (3)	0.035 (2)	0.031 (3)	0.003 (2)	0.000 (2)	0.003 (2)
C20	0.020 (3)	0.034 (2)	0.027 (2)	0.000 (2)	0.001 (2)	−0.0012 (19)
C21	0.031 (3)	0.032 (2)	0.035 (3)	0.004 (2)	−0.004 (2)	−0.003 (2)
C22	0.032 (3)	0.028 (2)	0.029 (2)	−0.002 (2)	−0.008 (2)	−0.004 (2)
C40	0.030 (3)	0.036 (2)	0.032 (3)	0.001 (2)	0.000 (2)	0.001 (2)
C23	0.048 (4)	0.048 (3)	0.036 (3)	−0.007 (3)	−0.005 (3)	0.002 (2)
C24	0.059 (4)	0.043 (3)	0.054 (4)	−0.009 (3)	−0.015 (3)	0.015 (3)
C25	0.056 (4)	0.035 (3)	0.068 (4)	0.005 (3)	−0.015 (3)	0.000 (3)
C26	0.049 (4)	0.042 (3)	0.054 (3)	0.008 (3)	−0.002 (3)	−0.011 (3)
C27	0.043 (3)	0.037 (2)	0.034 (3)	0.003 (2)	−0.004 (3)	−0.001 (2)
C28	0.027 (3)	0.034 (2)	0.025 (2)	0.006 (2)	0.000 (2)	−0.0059 (19)
C29	0.027 (3)	0.026 (2)	0.030 (2)	0.005 (2)	−0.002 (2)	−0.0034 (19)
C30	0.034 (3)	0.033 (2)	0.030 (2)	0.001 (2)	0.002 (2)	−0.002 (2)
C31	0.035 (3)	0.035 (2)	0.035 (3)	0.007 (2)	−0.006 (2)	−0.001 (2)
C32	0.046 (4)	0.049 (3)	0.054 (3)	0.000 (3)	−0.009 (3)	−0.018 (3)
C33	0.047 (4)	0.062 (3)	0.035 (3)	0.003 (3)	0.003 (3)	−0.008 (2)
C34	0.024 (3)	0.029 (2)	0.031 (2)	−0.005 (2)	−0.004 (2)	−0.003 (2)
C35	0.034 (3)	0.037 (2)	0.027 (2)	0.001 (2)	0.011 (2)	−0.005 (2)
C36	0.031 (3)	0.045 (3)	0.038 (3)	0.002 (2)	−0.003 (2)	−0.010 (2)
C37	0.044 (4)	0.045 (3)	0.082 (4)	0.010 (3)	0.004 (3)	−0.002 (3)
C38	0.052 (4)	0.078 (4)	0.040 (3)	0.015 (3)	0.000 (3)	−0.017 (3)
C39	0.058 (4)	0.098 (4)	0.050 (4)	0.013 (4)	0.006 (3)	−0.014 (3)
C41	0.024 (3)	0.036 (2)	0.031 (2)	0.002 (2)	−0.004 (2)	−0.001 (2)
C42	0.031 (3)	0.034 (2)	0.035 (3)	−0.001 (2)	−0.003 (2)	−0.003 (2)
C43	0.049 (4)	0.038 (3)	0.042 (3)	0.007 (3)	0.005 (3)	−0.006 (2)
C44	0.048 (4)	0.039 (3)	0.042 (3)	0.005 (3)	0.003 (3)	0.006 (2)
C45	0.082 (5)	0.047 (3)	0.070 (4)	0.001 (3)	0.025 (4)	0.005 (3)
C46	0.024 (3)	0.038 (3)	0.027 (2)	0.003 (2)	−0.005 (2)	0.002 (2)
C47	0.025 (3)	0.031 (2)	0.033 (2)	0.008 (2)	−0.001 (2)	−0.004 (2)
C48	0.032 (3)	0.036 (2)	0.036 (3)	0.009 (2)	−0.006 (2)	0.003 (2)
C49	0.044 (3)	0.037 (2)	0.033 (3)	0.004 (2)	−0.001 (3)	0.002 (2)
C50	0.065 (4)	0.049 (3)	0.059 (4)	−0.009 (3)	−0.006 (3)	0.010 (3)
C51	0.053 (4)	0.052 (3)	0.054 (3)	0.022 (3)	0.002 (3)	0.008 (3)
C52	0.031 (3)	0.037 (2)	0.027 (2)	0.007 (2)	−0.003 (2)	−0.008 (2)
C53	0.025 (3)	0.041 (2)	0.024 (2)	0.004 (2)	−0.001 (2)	−0.003 (2)
C54	0.028 (3)	0.045 (3)	0.032 (3)	0.003 (2)	−0.001 (2)	−0.005 (2)
C55	0.056 (4)	0.041 (3)	0.051 (3)	−0.005 (3)	0.000 (3)	0.006 (2)
C56	0.041 (3)	0.042 (3)	0.043 (3)	−0.005 (3)	0.000 (3)	−0.005 (2)
C57	0.027 (3)	0.043 (3)	0.025 (2)	0.001 (2)	0.008 (2)	0.002 (2)
O10	0.040 (2)	0.0381 (18)	0.060 (2)	−0.0069 (17)	−0.0121 (19)	0.0105 (16)
C58	0.085 (5)	0.062 (4)	0.083 (5)	−0.034 (4)	−0.002 (4)	0.017 (3)

### Geometric parameters (Å, °)

**Table:**

O1—C5	1.221 (5)	C23—H23	0.9500
O2—C10	1.228 (5)	C24—C25	1.372 (8)
O3—C19	1.233 (5)	C24—H24	0.9500
O4—C28	1.227 (5)	C25—C26	1.381 (7)
O5—C34	1.233 (5)	C25—H25	0.9500
O6—C40	1.228 (5)	C26—C27	1.380 (6)
O7—C46	1.227 (5)	C26—H26	0.9500
O8—C52	1.225 (6)	C27—H27	0.9500
O9—C57	1.246 (5)	C29—C34	1.526 (6)
N1—C57	1.337 (5)	C29—C30	1.528 (6)
N1—C4	1.465 (5)	C29—H29	1.0000
N1—C1	1.470 (5)	C30—C31	1.531 (6)
N2—C5	1.355 (6)	C30—H30A	0.9900
N2—C6	1.465 (5)	C30—H30B	0.9900
N2—C9	1.482 (5)	C31—C32	1.512 (6)
N3—C10	1.346 (5)	C31—C33	1.520 (7)
N3—C11	1.457 (6)	C31—H31	1.0000
N3—H3	0.8800	C32—H32A	0.9800
N4—C19	1.337 (6)	C32—H32B	0.9800
N4—C20	1.464 (5)	C32—H32C	0.9800
N4—H4	0.8800	C33—H33A	0.9800
N5—C28	1.349 (5)	C33—H33B	0.9800
N5—C29	1.458 (5)	C33—H33C	0.9800
N5—H5	0.8800	C35—C36	1.546 (6)
N6—C34	1.346 (5)	C35—H35	1.0000
N6—C35	1.470 (5)	C36—C37	1.520 (6)
N6—H6	0.8800	C36—C38	1.543 (6)
N7—C40	1.346 (6)	C36—H36	1.0000
N7—C41	1.460 (6)	C37—H37A	0.9800
N7—H7	0.8800	C37—H37B	0.9800
N8—C46	1.348 (5)	C37—H37C	0.9800
N8—C47	1.459 (5)	C38—C39	1.502 (8)
N8—H8	0.8800	C38—H38A	0.9900
N9—C52	1.350 (6)	C38—H38B	0.9900
N9—C53	1.458 (5)	C39—H39A	0.9800
N9—H9	0.8800	C39—H39B	0.9800
C1—C5	1.527 (6)	C39—H39C	0.9800
C1—C2	1.537 (6)	C41—C42	1.527 (6)
C1—H1	1.0000	C41—C46	1.529 (6)
C2—C3	1.524 (7)	C41—H41	1.0000
C2—H2A	0.9900	C42—C44	1.526 (6)
C2—H2B	0.9900	C42—C43	1.535 (6)
C3—C4	1.517 (6)	C42—H42	1.0000
C3—H3A	0.9900	C43—H43A	0.9800
C3—H3B	0.9900	C43—H43B	0.9800
C4—H4A	0.9900	C43—H43C	0.9800
C4—H4B	0.9900	C44—C45	1.527 (6)
C6—C10	1.525 (6)	C44—H44A	0.9900
C6—C7	1.539 (6)	C44—H44B	0.9900
C6—H6A	1.0000	C45—H45A	0.9800
C7—C8	1.519 (6)	C45—H45B	0.9800
C7—H7A	0.9900	C45—H45C	0.9800
C7—H7B	0.9900	C47—C48	1.533 (6)
C8—C9	1.513 (7)	C47—C52	1.544 (6)
C8—H8A	0.9900	C47—H47	1.0000
C8—H8B	0.9900	C48—C49	1.523 (6)
C9—H9A	0.9900	C48—H48A	0.9900
C9—H9B	0.9900	C48—H48B	0.9900
C11—C19	1.530 (6)	C49—C50	1.533 (7)
C11—C12	1.534 (6)	C49—C51	1.535 (7)
C11—H11	1.0000	C49—H49	1.0000
C12—C13	1.510 (6)	C50—H50A	0.9800
C12—H12A	0.9900	C50—H50B	0.9800
C12—H12B	0.9900	C50—H50C	0.9800
C13—C18	1.376 (7)	C51—H51A	0.9800
C13—C14	1.388 (7)	C51—H51B	0.9800
C14—C15	1.397 (7)	C51—H51C	0.9800
C14—H14	0.9500	C53—C57	1.523 (6)
C15—C16	1.369 (8)	C53—C54	1.537 (6)
C15—H15	0.9500	C53—H53	1.0000
C16—C17	1.374 (8)	C54—C56	1.531 (6)
C16—H16	0.9500	C54—C55	1.537 (6)
C17—C18	1.386 (7)	C54—H54	1.0000
C17—H17	0.9500	C55—H55A	0.9800
C18—H18	0.9500	C55—H55B	0.9800
C20—C21	1.521 (6)	C55—H55C	0.9800
C20—C28	1.530 (6)	C56—H56A	0.9800
C20—H20	1.0000	C56—H56B	0.9800
C21—C22	1.514 (6)	C56—H56C	0.9800
C21—H21A	0.9900	O10—C58	1.414 (6)
C21—H21B	0.9900	O10—H10	0.8400
C22—C23	1.383 (6)	C58—H58A	0.9800
C22—C27	1.388 (6)	C58—H58B	0.9800
C40—C35	1.525 (7)	C58—H58C	0.9800
C23—C24	1.389 (7)		
			
C57—N1—C4	127.1 (4)	C29—C30—H30A	108.1
C57—N1—C1	120.3 (4)	C31—C30—H30A	108.1
C4—N1—C1	112.5 (3)	C29—C30—H30B	108.1
C5—N2—C6	127.3 (4)	C31—C30—H30B	108.1
C5—N2—C9	119.0 (4)	H30A—C30—H30B	107.3
C6—N2—C9	111.8 (4)	C32—C31—C33	110.7 (4)
C10—N3—C11	121.7 (4)	C32—C31—C30	108.3 (4)
C10—N3—H3	119.2	C33—C31—C30	113.2 (4)
C11—N3—H3	119.2	C32—C31—H31	108.2
C19—N4—C20	122.7 (4)	C33—C31—H31	108.2
C19—N4—H4	118.6	C30—C31—H31	108.2
C20—N4—H4	118.6	C31—C32—H32A	109.5
C28—N5—C29	119.9 (4)	C31—C32—H32B	109.5
C28—N5—H5	120.1	H32A—C32—H32B	109.5
C29—N5—H5	120.1	C31—C32—H32C	109.5
C34—N6—C35	122.1 (4)	H32A—C32—H32C	109.5
C34—N6—H6	119.0	H32B—C32—H32C	109.5
C35—N6—H6	119.0	C31—C33—H33A	109.5
C40—N7—C41	121.9 (4)	C31—C33—H33B	109.5
C40—N7—H7	119.0	H33A—C33—H33B	109.5
C41—N7—H7	119.0	C31—C33—H33C	109.5
C46—N8—C47	120.3 (4)	H33A—C33—H33C	109.5
C46—N8—H8	119.8	H33B—C33—H33C	109.5
C47—N8—H8	119.8	O5—C34—N6	122.6 (4)
C52—N9—C53	123.5 (4)	O5—C34—C29	120.3 (4)
C52—N9—H9	118.2	N6—C34—C29	117.1 (4)
C53—N9—H9	118.2	N6—C35—C40	113.2 (4)
N1—C1—C5	110.9 (4)	N6—C35—C36	109.3 (4)
N1—C1—C2	102.4 (3)	C40—C35—C36	112.0 (3)
C5—C1—C2	111.0 (4)	N6—C35—H35	107.3
N1—C1—H1	110.8	C40—C35—H35	107.3
C5—C1—H1	110.8	C36—C35—H35	107.3
C2—C1—H1	110.8	C37—C36—C38	113.5 (4)
C3—C2—C1	104.1 (4)	C37—C36—C35	113.1 (4)
C3—C2—H2A	110.9	C38—C36—C35	110.1 (4)
C1—C2—H2A	110.9	C37—C36—H36	106.5
C3—C2—H2B	110.9	C38—C36—H36	106.5
C1—C2—H2B	110.9	C35—C36—H36	106.5
H2A—C2—H2B	109.0	C36—C37—H37A	109.5
C4—C3—C2	102.8 (4)	C36—C37—H37B	109.5
C4—C3—H3A	111.2	H37A—C37—H37B	109.5
C2—C3—H3A	111.2	C36—C37—H37C	109.5
C4—C3—H3B	111.2	H37A—C37—H37C	109.5
C2—C3—H3B	111.2	H37B—C37—H37C	109.5
H3A—C3—H3B	109.1	C39—C38—C36	114.0 (5)
N1—C4—C3	103.5 (4)	C39—C38—H38A	108.7
N1—C4—H4A	111.1	C36—C38—H38A	108.7
C3—C4—H4A	111.1	C39—C38—H38B	108.7
N1—C4—H4B	111.1	C36—C38—H38B	108.7
C3—C4—H4B	111.1	H38A—C38—H38B	107.6
H4A—C4—H4B	109.0	C38—C39—H39A	109.5
O1—C5—N2	122.3 (4)	C38—C39—H39B	109.5
O1—C5—C1	121.6 (4)	H39A—C39—H39B	109.5
N2—C5—C1	116.1 (4)	C38—C39—H39C	109.5
N2—C6—C10	115.6 (3)	H39A—C39—H39C	109.5
N2—C6—C7	102.9 (4)	H39B—C39—H39C	109.5
C10—C6—C7	110.5 (4)	N7—C41—C42	112.7 (4)
N2—C6—H6A	109.2	N7—C41—C46	112.4 (3)
C10—C6—H6A	109.2	C42—C41—C46	111.9 (4)
C7—C6—H6A	109.2	N7—C41—H41	106.4
C8—C7—C6	103.1 (4)	C42—C41—H41	106.4
C8—C7—H7A	111.2	C46—C41—H41	106.4
C6—C7—H7A	111.2	C44—C42—C41	111.7 (3)
C8—C7—H7B	111.2	C44—C42—C43	111.4 (4)
C6—C7—H7B	111.2	C41—C42—C43	111.1 (4)
H7A—C7—H7B	109.1	C44—C42—H42	107.4
C9—C8—C7	104.4 (4)	C41—C42—H42	107.4
C9—C8—H8A	110.9	C43—C42—H42	107.4
C7—C8—H8A	110.9	C42—C43—H43A	109.5
C9—C8—H8B	110.9	C42—C43—H43B	109.5
C7—C8—H8B	110.9	H43A—C43—H43B	109.5
H8A—C8—H8B	108.9	C42—C43—H43C	109.5
N2—C9—C8	103.7 (4)	H43A—C43—H43C	109.5
N2—C9—H9A	111.0	H43B—C43—H43C	109.5
C8—C9—H9A	111.0	C42—C44—C45	114.1 (4)
N2—C9—H9B	111.0	C42—C44—H44A	108.7
C8—C9—H9B	111.0	C45—C44—H44A	108.7
H9A—C9—H9B	109.0	C42—C44—H44B	108.7
O2—C10—N3	123.9 (4)	C45—C44—H44B	108.7
O2—C10—C6	118.1 (4)	H44A—C44—H44B	107.6
N3—C10—C6	118.0 (4)	C44—C45—H45A	109.5
N3—C11—C19	112.9 (4)	C44—C45—H45B	109.5
N3—C11—C12	111.4 (4)	H45A—C45—H45B	109.5
C19—C11—C12	111.5 (4)	C44—C45—H45C	109.5
N3—C11—H11	106.9	H45A—C45—H45C	109.5
C19—C11—H11	106.9	H45B—C45—H45C	109.5
C12—C11—H11	106.9	O7—C46—N8	121.9 (4)
C13—C12—C11	112.0 (3)	O7—C46—C41	121.4 (4)
C13—C12—H12A	109.2	N8—C46—C41	116.7 (4)
C11—C12—H12A	109.2	N8—C47—C48	115.2 (4)
C13—C12—H12B	109.2	N8—C47—C52	110.8 (3)
C11—C12—H12B	109.2	C48—C47—C52	112.9 (4)
H12A—C12—H12B	107.9	N8—C47—H47	105.7
C18—C13—C14	118.3 (4)	C48—C47—H47	105.7
C18—C13—C12	122.9 (5)	C52—C47—H47	105.7
C14—C13—C12	118.8 (4)	C49—C48—C47	114.6 (4)
C13—C14—C15	120.3 (5)	C49—C48—H48A	108.6
C13—C14—H14	119.9	C47—C48—H48A	108.6
C15—C14—H14	119.9	C49—C48—H48B	108.6
C16—C15—C14	120.5 (6)	C47—C48—H48B	108.6
C16—C15—H15	119.7	H48A—C48—H48B	107.6
C14—C15—H15	119.7	C48—C49—C50	111.6 (4)
C15—C16—C17	119.4 (5)	C48—C49—C51	109.6 (4)
C15—C16—H16	120.3	C50—C49—C51	110.5 (4)
C17—C16—H16	120.3	C48—C49—H49	108.4
C16—C17—C18	120.4 (6)	C50—C49—H49	108.4
C16—C17—H17	119.8	C51—C49—H49	108.4
C18—C17—H17	119.8	C49—C50—H50A	109.5
C13—C18—C17	121.1 (6)	C49—C50—H50B	109.5
C13—C18—H18	119.4	H50A—C50—H50B	109.5
C17—C18—H18	119.4	C49—C50—H50C	109.5
O3—C19—N4	121.7 (4)	H50A—C50—H50C	109.5
O3—C19—C11	119.2 (4)	H50B—C50—H50C	109.5
N4—C19—C11	119.0 (4)	C49—C51—H51A	109.5
N4—C20—C21	111.5 (4)	C49—C51—H51B	109.5
N4—C20—C28	111.0 (3)	H51A—C51—H51B	109.5
C21—C20—C28	111.1 (4)	C49—C51—H51C	109.5
N4—C20—H20	107.7	H51A—C51—H51C	109.5
C21—C20—H20	107.7	H51B—C51—H51C	109.5
C28—C20—H20	107.7	O8—C52—N9	123.2 (5)
C22—C21—C20	113.0 (4)	O8—C52—C47	121.8 (4)
C22—C21—H21A	109.0	N9—C52—C47	114.9 (4)
C20—C21—H21A	109.0	N9—C53—C57	106.7 (3)
C22—C21—H21B	109.0	N9—C53—C54	113.3 (3)
C20—C21—H21B	109.0	C57—C53—C54	113.0 (4)
H21A—C21—H21B	107.8	N9—C53—H53	107.9
C23—C22—C27	118.9 (4)	C57—C53—H53	107.9
C23—C22—C21	121.4 (4)	C54—C53—H53	107.9
C27—C22—C21	119.6 (4)	C56—C54—C53	110.0 (4)
O6—C40—N7	122.9 (5)	C56—C54—C55	109.5 (4)
O6—C40—C35	119.4 (4)	C53—C54—C55	110.4 (4)
N7—C40—C35	117.6 (4)	C56—C54—H54	108.9
C22—C23—C24	119.9 (5)	C53—C54—H54	108.9
C22—C23—H23	120.0	C55—C54—H54	108.9
C24—C23—H23	120.0	C54—C55—H55A	109.5
C25—C24—C23	120.6 (5)	C54—C55—H55B	109.5
C25—C24—H24	119.7	H55A—C55—H55B	109.5
C23—C24—H24	119.7	C54—C55—H55C	109.5
C24—C25—C26	119.9 (5)	H55A—C55—H55C	109.5
C24—C25—H25	120.1	H55B—C55—H55C	109.5
C26—C25—H25	120.1	C54—C56—H56A	109.5
C27—C26—C25	119.7 (5)	C54—C56—H56B	109.5
C27—C26—H26	120.2	H56A—C56—H56B	109.5
C25—C26—H26	120.2	C54—C56—H56C	109.5
C26—C27—C22	121.0 (5)	H56A—C56—H56C	109.5
C26—C27—H27	119.5	H56B—C56—H56C	109.5
C22—C27—H27	119.5	O9—C57—N1	120.9 (4)
O4—C28—N5	121.7 (4)	O9—C57—C53	122.4 (4)
O4—C28—C20	123.0 (4)	N1—C57—C53	116.7 (4)
N5—C28—C20	115.3 (4)	C58—O10—H10	109.5
N5—C29—C34	112.1 (3)	O10—C58—H58A	109.5
N5—C29—C30	110.9 (4)	O10—C58—H58B	109.5
C34—C29—C30	110.4 (3)	H58A—C58—H58B	109.5
N5—C29—H29	107.7	O10—C58—H58C	109.5
C34—C29—H29	107.7	H58A—C58—H58C	109.5
C30—C29—H29	107.7	H58B—C58—H58C	109.5
C29—C30—C31	116.6 (4)		
			
C57—N1—C1—C5	−66.9 (5)	C29—N5—C28—O4	3.8 (6)
C4—N1—C1—C5	109.6 (4)	C29—N5—C28—C20	−179.0 (3)
C57—N1—C1—C2	174.6 (4)	N4—C20—C28—O4	−118.6 (4)
C4—N1—C1—C2	−8.9 (5)	C21—C20—C28—O4	6.1 (6)
N1—C1—C2—C3	29.3 (4)	N4—C20—C28—N5	64.3 (5)
C5—C1—C2—C3	−89.1 (5)	C21—C20—C28—N5	−171.0 (4)
C1—C2—C3—C4	−38.9 (5)	C28—N5—C29—C34	−62.7 (5)
C57—N1—C4—C3	161.1 (4)	C28—N5—C29—C30	173.4 (4)
C1—N1—C4—C3	−15.1 (5)	N5—C29—C30—C31	−56.4 (5)
C2—C3—C4—N1	32.7 (5)	C34—C29—C30—C31	178.7 (4)
C6—N2—C5—O1	−170.5 (4)	C29—C30—C31—C32	−175.6 (4)
C9—N2—C5—O1	−7.6 (7)	C29—C30—C31—C33	−52.4 (5)
C6—N2—C5—C1	8.1 (6)	C35—N6—C34—O5	3.3 (7)
C9—N2—C5—C1	170.9 (4)	C35—N6—C34—C29	−177.3 (4)
N1—C1—C5—O1	−16.6 (6)	N5—C29—C34—O5	145.4 (4)
C2—C1—C5—O1	96.5 (5)	C30—C29—C34—O5	−90.4 (5)
N1—C1—C5—N2	164.8 (4)	N5—C29—C34—N6	−33.9 (6)
C2—C1—C5—N2	−82.1 (5)	C30—C29—C34—N6	90.3 (4)
C5—N2—C6—C10	−91.5 (5)	C34—N6—C35—C40	−57.2 (5)
C9—N2—C6—C10	104.6 (4)	C34—N6—C35—C36	177.2 (4)
C5—N2—C6—C7	148.0 (4)	O6—C40—C35—N6	152.2 (4)
C9—N2—C6—C7	−15.9 (5)	N7—C40—C35—N6	−30.8 (5)
N2—C6—C7—C8	32.5 (5)	O6—C40—C35—C36	−83.6 (5)
C10—C6—C7—C8	−91.4 (5)	N7—C40—C35—C36	93.3 (5)
C6—C7—C8—C9	−37.8 (5)	N6—C35—C36—C37	60.5 (5)
C5—N2—C9—C8	−172.7 (4)	C40—C35—C36—C37	−65.8 (5)
C6—N2—C9—C8	−7.3 (5)	N6—C35—C36—C38	−171.4 (4)
C7—C8—C9—N2	28.0 (5)	C40—C35—C36—C38	62.3 (5)
C11—N3—C10—O2	2.7 (7)	C37—C36—C38—C39	−62.0 (7)
C11—N3—C10—C6	−176.8 (4)	C35—C36—C38—C39	170.1 (5)
N2—C6—C10—O2	172.7 (4)	C40—N7—C41—C42	119.7 (4)
C7—C6—C10—O2	−71.0 (5)	C40—N7—C41—C46	−112.7 (4)
N2—C6—C10—N3	−7.7 (6)	N7—C41—C42—C44	55.7 (5)
C7—C6—C10—N3	108.6 (4)	C46—C41—C42—C44	−72.1 (5)
C10—N3—C11—C19	−103.2 (5)	N7—C41—C42—C43	−69.5 (5)
C10—N3—C11—C12	130.5 (4)	C46—C41—C42—C43	162.8 (4)
N3—C11—C12—C13	−60.8 (5)	C41—C42—C44—C45	161.9 (4)
C19—C11—C12—C13	172.1 (4)	C43—C42—C44—C45	−73.2 (6)
C11—C12—C13—C18	109.8 (5)	C47—N8—C46—O7	−4.4 (6)
C11—C12—C13—C14	−70.3 (6)	C47—N8—C46—C41	175.4 (4)
C18—C13—C14—C15	−1.6 (7)	N7—C41—C46—O7	−158.5 (4)
C12—C13—C14—C15	178.5 (4)	C42—C41—C46—O7	−30.6 (6)
C13—C14—C15—C16	1.7 (7)	N7—C41—C46—N8	21.7 (5)
C14—C15—C16—C17	−1.3 (8)	C42—C41—C46—N8	149.6 (4)
C15—C16—C17—C18	1.0 (8)	C46—N8—C47—C48	−72.2 (5)
C14—C13—C18—C17	1.3 (7)	C46—N8—C47—C52	57.5 (5)
C12—C13—C18—C17	−178.9 (5)	N8—C47—C48—C49	−134.0 (4)
C16—C17—C18—C13	−1.0 (8)	C52—C47—C48—C49	97.3 (5)
C20—N4—C19—O3	1.1 (7)	C47—C48—C49—C50	78.3 (5)
C20—N4—C19—C11	−177.2 (4)	C47—C48—C49—C51	−159.0 (4)
N3—C11—C19—O3	134.6 (4)	C53—N9—C52—O8	−2.6 (7)
C12—C11—C19—O3	−99.1 (5)	C53—N9—C52—C47	173.5 (3)
N3—C11—C19—N4	−47.0 (5)	N8—C47—C52—O8	−142.7 (4)
C12—C11—C19—N4	79.3 (5)	C48—C47—C52—O8	−11.7 (6)
C19—N4—C20—C21	151.1 (4)	N8—C47—C52—N9	41.1 (5)
C19—N4—C20—C28	−84.4 (5)	C48—C47—C52—N9	172.1 (4)
N4—C20—C21—C22	−76.0 (5)	C52—N9—C53—C57	−125.3 (4)
C28—C20—C21—C22	159.6 (4)	C52—N9—C53—C54	109.7 (5)
C20—C21—C22—C23	106.8 (5)	N9—C53—C54—C56	−56.7 (5)
C20—C21—C22—C27	−73.3 (5)	C57—C53—C54—C56	−178.2 (4)
C41—N7—C40—O6	−4.6 (6)	N9—C53—C54—C55	−177.7 (4)
C41—N7—C40—C35	178.5 (3)	C57—C53—C54—C55	60.8 (5)
C27—C22—C23—C24	−0.6 (7)	C4—N1—C57—O9	−178.1 (4)
C21—C22—C23—C24	179.3 (4)	C1—N1—C57—O9	−2.2 (6)
C22—C23—C24—C25	0.8 (8)	C4—N1—C57—C53	4.3 (6)
C23—C24—C25—C26	−0.4 (8)	C1—N1—C57—C53	−179.8 (4)
C24—C25—C26—C27	−0.2 (8)	N9—C53—C57—O9	−103.7 (5)
C25—C26—C27—C22	0.4 (8)	C54—C53—C57—O9	21.5 (6)
C23—C22—C27—C26	−0.1 (7)	N9—C53—C57—N1	73.9 (5)
C21—C22—C27—C26	−179.9 (4)	C54—C53—C57—N1	−160.9 (4)

### Hydrogen-bond geometry (Å, °)

**Table:**

*D*—H···*A*	*D*—H	H···*A*	*D*···*A*	*D*—H···*A*
N3—H3···O10	0.88	2.09	2.912 (5)	154
N4—H4···O10	0.88	2.28	3.139 (5)	166
N4—H4···O9	0.88	2.51	3.103 (5)	125
N5—H5···O3	0.88	2.18	2.935 (5)	143
N6—H6···O8^i^	0.88	2.49	3.208 (5)	139
N7—H7···O4	0.88	2.50	3.266 (5)	145
N8—H8···O5	0.88	2.11	2.971 (5)	165
N9—H9···O4	0.88	2.13	2.975 (5)	161
O10—H10···O9	0.84	1.91	2.698 (4)	156

Symmetry codes: (i) *x*+1, *y*, *z*.
